# National HIV Testing Day — June 27, 2018

**DOI:** 10.15585/mmwr.mm6724a1

**Published:** 2018-06-22

National HIV Testing Day, June 27, highlights the importance of testing in detecting, treating, and preventing human immunodeficiency virus (HIV) infection. Awareness of HIV infection through HIV testing is the first step to prevention, health care, and social services that improve life quality and length of survival (*1*).

Health care providers and others providing HIV testing can reduce HIV-related adverse health outcomes and risk for HIV transmission by implementing routine and targeted testing to decrease diagnosis delays (*2*). In this issue, an analysis of national population-based survey data collected during 2006–2016 found that persons with higher risk for HIV in the past year did not achieve CDC’s recommended frequency of at least annual screening and the median time between tests did not change (*3*). Health care providers and public health practitioners need to intensify efforts to routinely screen all patients for HIV infection, and to identify persons with ongoing risk and ensure that they are engaged in annual screening for HIV infection.

Additional information on National HIV Testing Day is available at https://www.cdc.gov/features/HIVtesting. Basic testing information for the public is available at https://www.cdc.gov/hiv/basics/testing.html. Additional information on HIV testing for health professionals is available at https://www.cdc.gov/hiv/testing. CDC’s guidelines for HIV testing of serum and plasma specimens are available at https://www.cdc.gov/hiv/guidelines/testing.html.
